# Supplementary material for: Genome-Wide Analysis Identifies Candidate Genes Encoding Feather Color in Ducks
Source: Genes (Basel). 2022 Jul 14;13(7):1249. doi: 10.3390/genes13071249 (PMC9317390; doi:10.3390/genes13071249)
Supplement: Supplementary file 1 [file genes-13-01249-s001.zip › genes-1787452-supplementary.pdf]

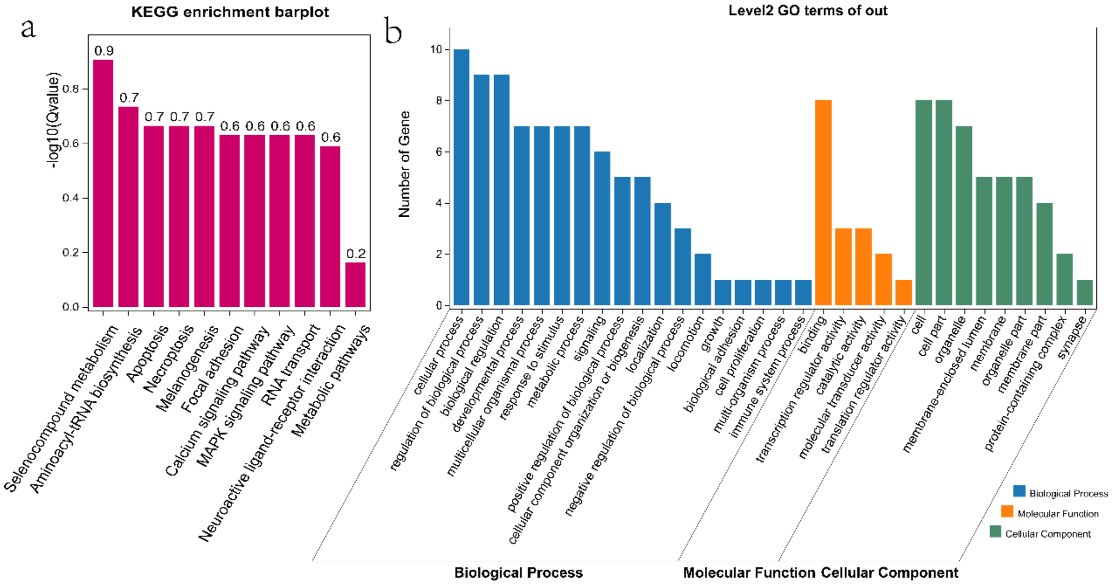

Figure S1. KEGG (a) and GO (b) enrichment analysis of black plumage associate genes.

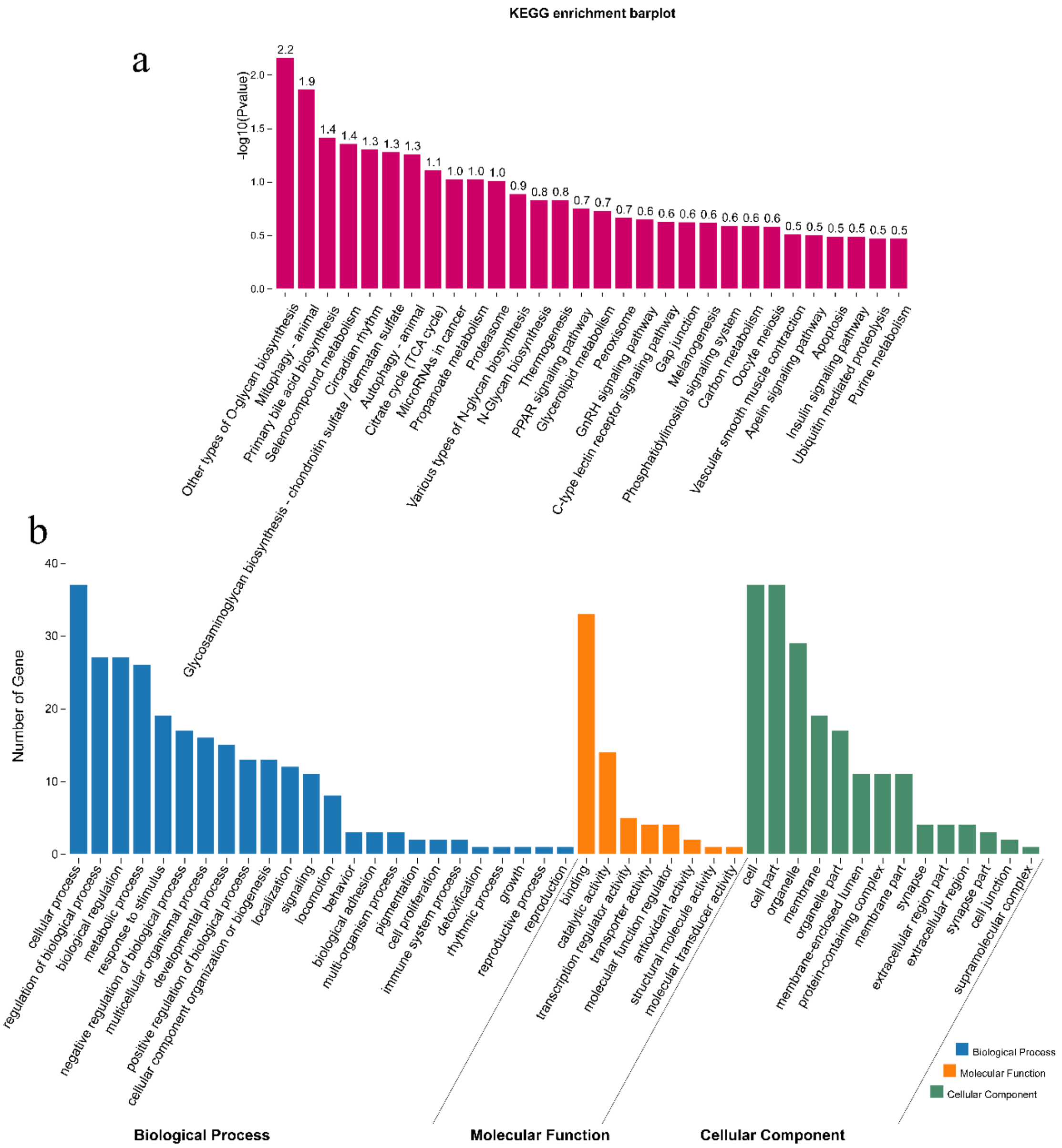

Figure S2. KEGG (a) and GO (b) enrichment analysis of white plumage associate genes.

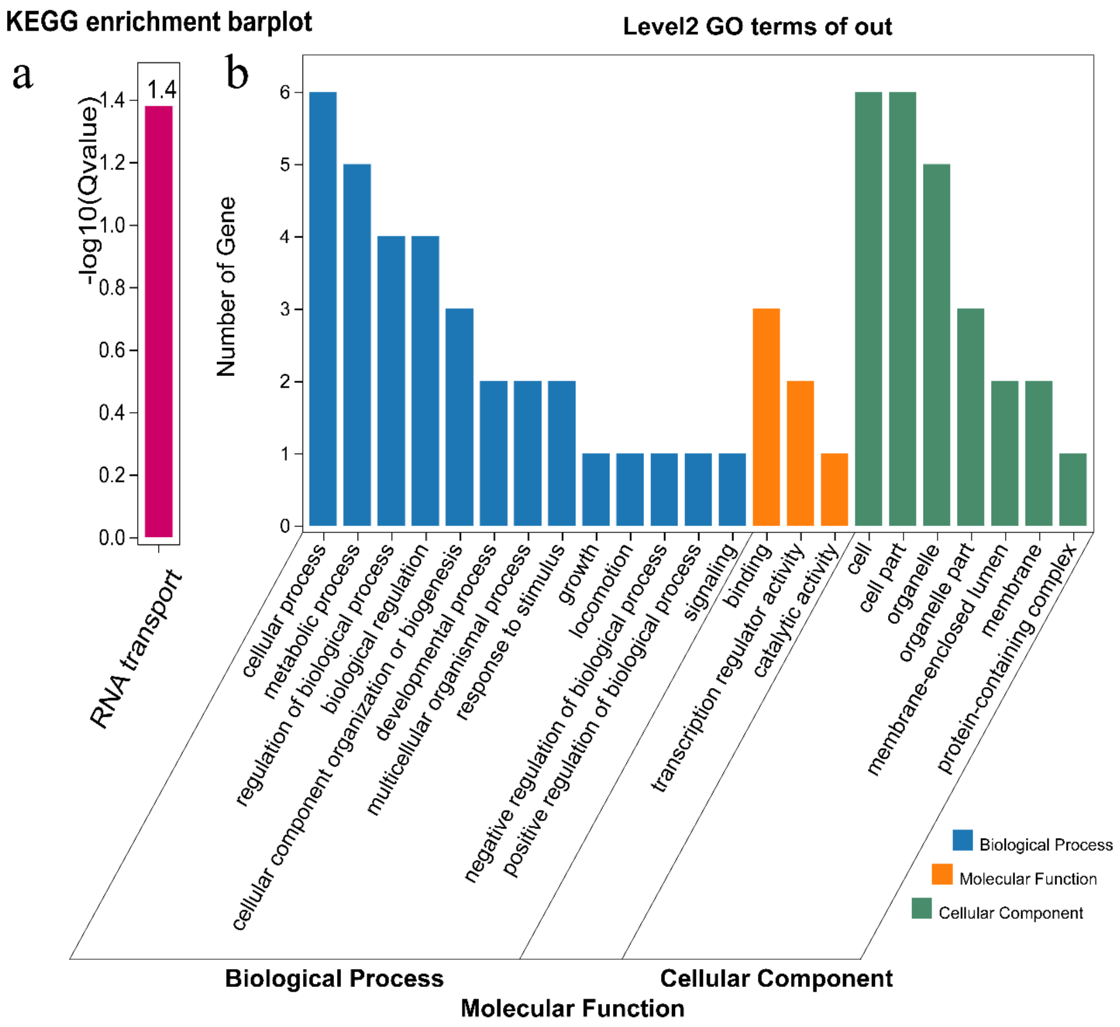

Figure S3. KEGG (a) and GO (b) enrichment analysis of spot plumage associate genes.

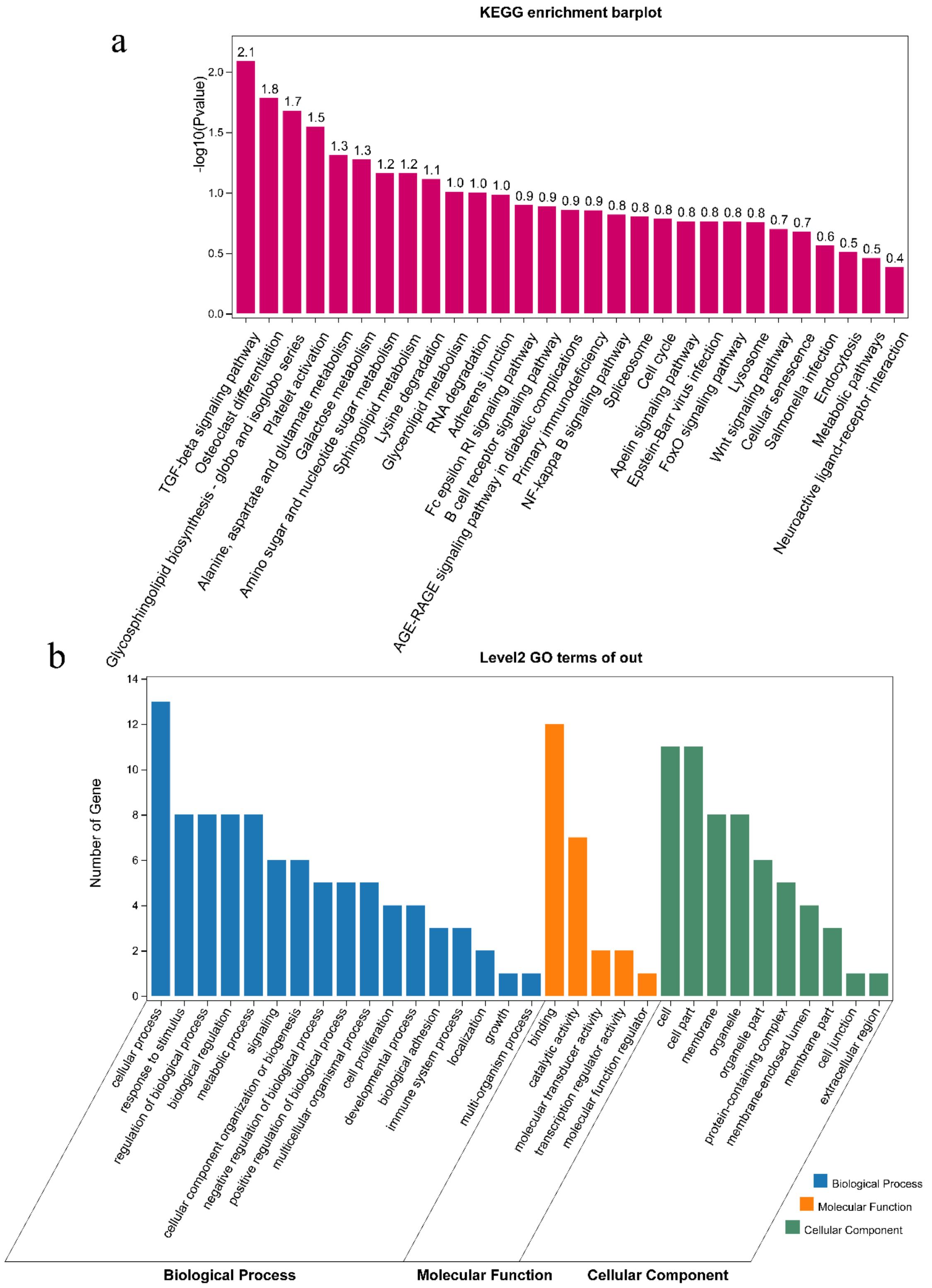

Figure S4. KEGG (a) and GO (b) enrichment analysis of gray plumage associate genes.

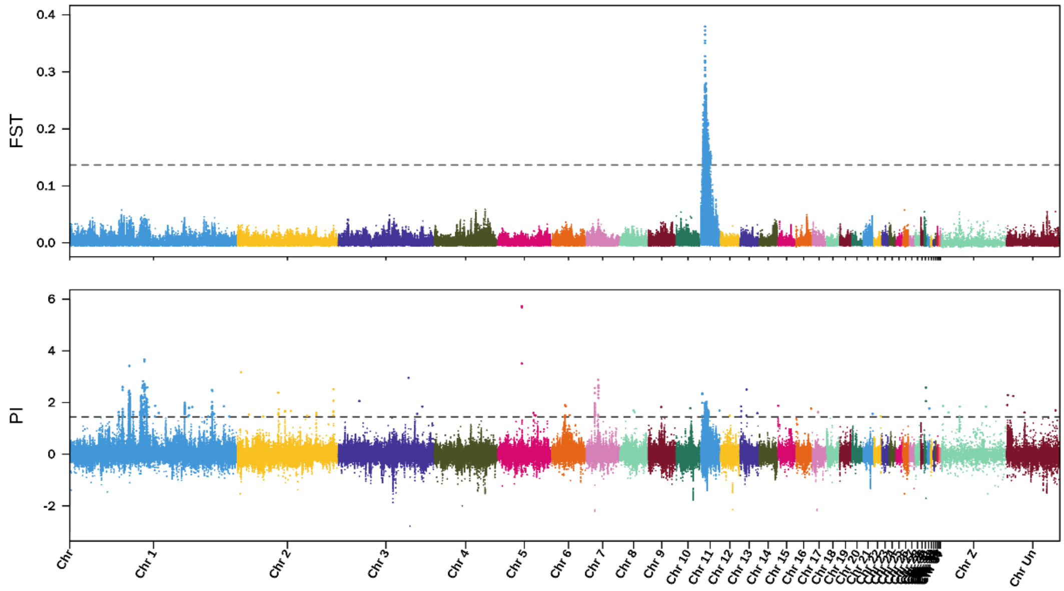

Figure S5. Genome-wide selective signals across white plumage duck based on global F_{ST} (top) and log2 π ratio (bottom).

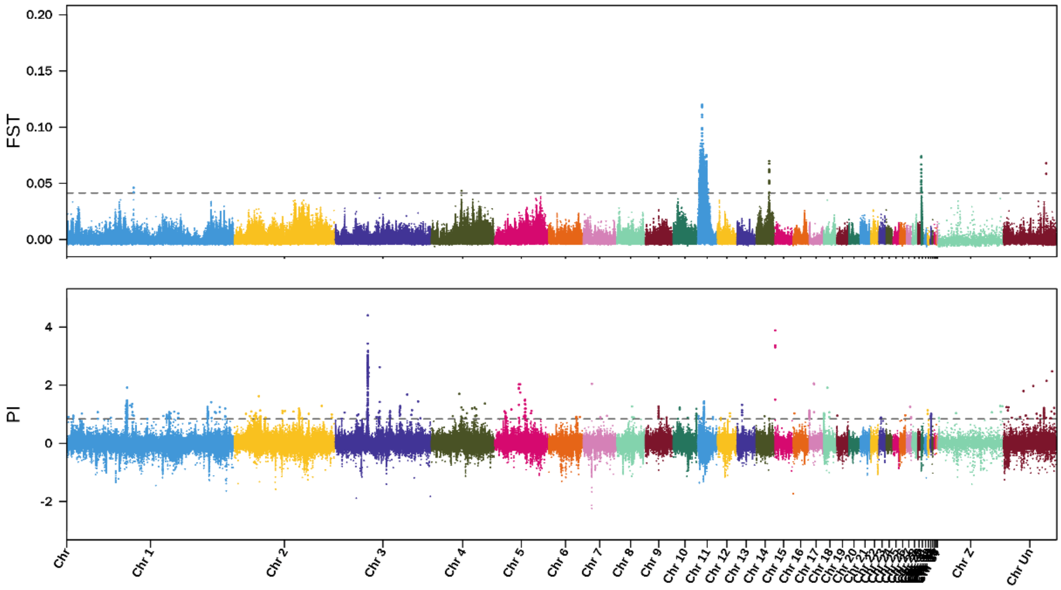

Figure S6. Genome-wide selective signals across spot plumage duck based on global F_{ST} (top) and log2 π ratio (bottom).

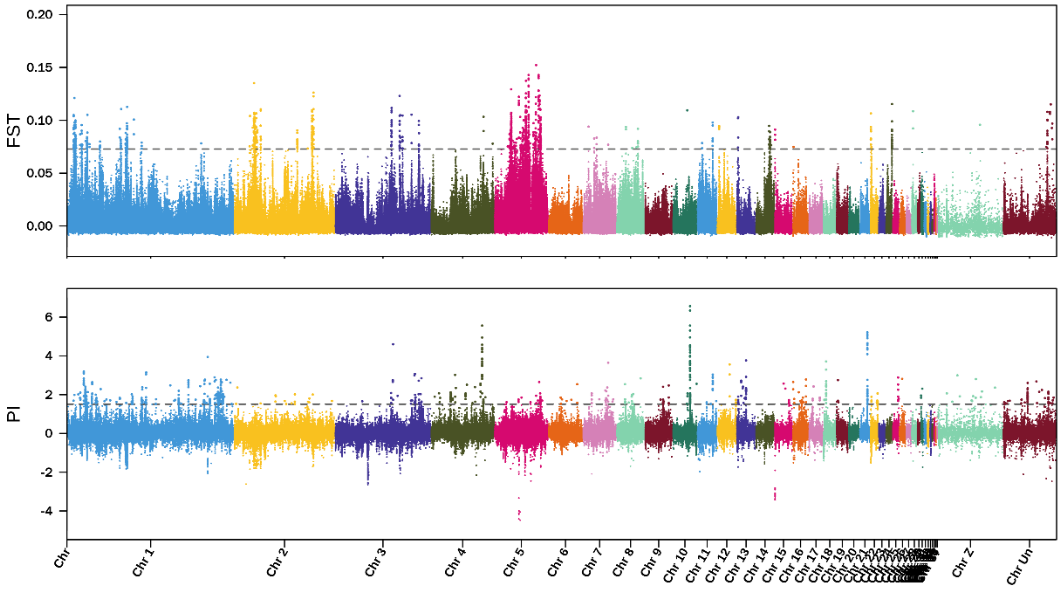

Figure S7. Genome-wide selective signals across gray plumage duck based on global F_{ST} (top) and log2 π ratio (bottom).

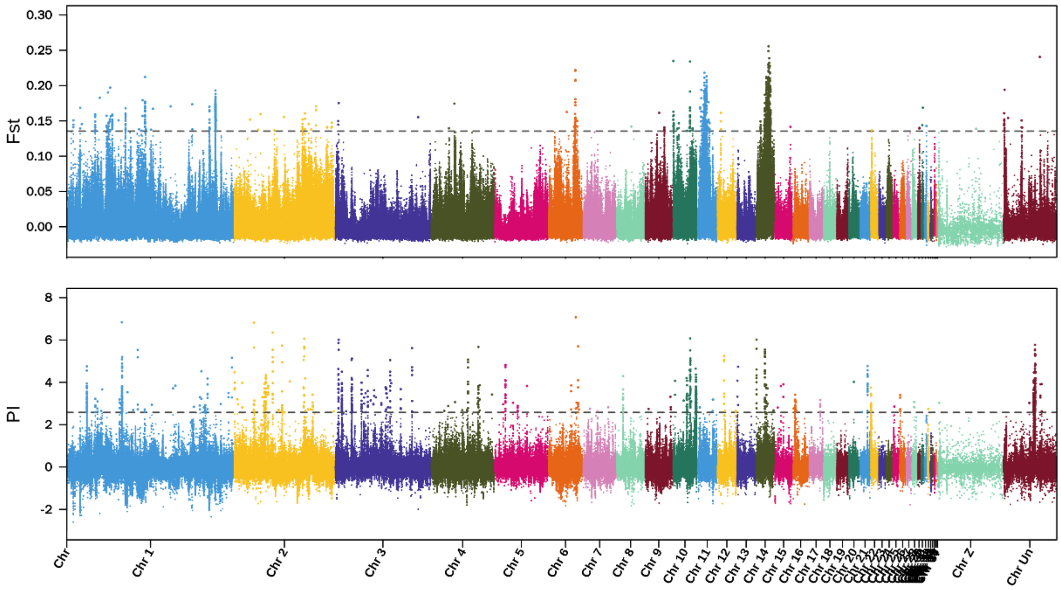

Figure S8. Genome-wide selective signals across black plumage duck based on global F_{ST} (top) and log2 π ratio (bottom).
